# Essentials of Surgery, Together with a Full Description of the Handkerchief and Roller Bandage, Arranged in the Form of Questions and Answers

**Published:** 1900-11

**Authors:** 


Essentials of Surgery, together with a full description of the Handkerchief and Roller Bandage, arranged in the form of questions and answers. Prepared especially for students of medicine. By Edward Martin, A. M., M. D., Clinical Professor of Genito-Urinary Diseases in the University of Pennsylvania. Illustrated. Seventh edition, revised and enlarged, with an appendix containing full direction and prescriptions for the preparations of the various materials in antiseptic surgery; also several hundred receipts covering the medical treatment of surgical affections. Pages, 342; price, $1.00. Philadelphia: W. B. Saunders, 925 Walnut Street. 1900.

This book, like the others of Saunders' Question Compend Series, is well gotten up, and is quite the thing for students who desire to cover a large field in a short time.

